# A Case of Coil-Assisted Retrograde Transvenous Obliteration to Treat Colonic Varices

**DOI:** 10.7759/cureus.63850

**Published:** 2024-07-04

**Authors:** Noor Hassan, Mir Zulqarnain, Abbas Bader, Maaz Hassan, Islam Mohamed, Travis Brown, Kavita Jadhav, Hassan Ghoz

**Affiliations:** 1 Internal Medicine, University of Missouri Kansas City School of Medicine, Kansas City, USA; 2 Gastroenterology and Hepatology, University of Missouri Kansas City School of Medicine, Kansas City, USA; 3 Radiology, University of Kansas School of Medicine, Kansas City, USA; 4 Interventional Radiology, University of Missouri Kansas City School of Medicine, Kansas City, USA

**Keywords:** colonic varices, varices, lower gastrointestinal bleeding, balloon-occluded retrograde transvenous obliteration (brto), coil-assisted retrograde transvenous obliteration (crto), interventional radiology guided embolization, decompensated liver cirrhosis, ectopic varices, coil embolization

## Abstract

Colonic variceal bleeding is a rare cause of lower gastrointestinal (GI) bleeding, which carries a high mortality rate. Due to limited data, the optimal management of colonic variceal bleeding is not known. Coil-assisted retrograde transvenous obliteration (CARTO) has been shown to be very effective in managing non-esophageal variceal bleeding, but only a few cases demonstrate its effectiveness in treating colonic variceal bleeding. Here we present a case of colonic variceal bleeding treated with CARTO in order to expand on the limited body of evidence showing its efficacy in effectively treating this rare cause of life-threatening GI bleeding.

## Introduction

Colonic variceal bleeding is an extremely rare form of lower gastrointestinal (GI) bleeding that, if left untreated, can have fatal consequences, with a mortality rate of up to 25% [1,2]. Commonly, colonic varices are found in the rectosigmoid region or the cecum [3]. The most common cause of colonic variceal bleeding is portal hypertension; however, other causes may include mesenteric vein thrombosis or compression, congestive heart failure, and pancreatitis with splenic vein thrombosis and adhesions [4]. Treatment of colonic varices is non-standardized due to their rarity.

Coil-assisted retrograde transvenous obliteration (CARTO) is a modified procedure of balloon-occluded retrograde transvenous obliteration (BRTO), which implements the use of gel foam and coils. Like BRTO, CARTO has been shown to be very effective in treating variceal bleeding other than esophageal, including gastric and duodenal [2]. Lee et al. document a retrospective study with 20 patients with portal hypertensive non-esophageal variceal bleeding treated with CARTO who suffered no variceal re-bleeding [5]. However, this study did not include any patients with colonic variceal bleeding. We report a case of colonic varices treated with coil-assisted retrograde transvenous obliteration.

## Case presentation

**Figure 1:**
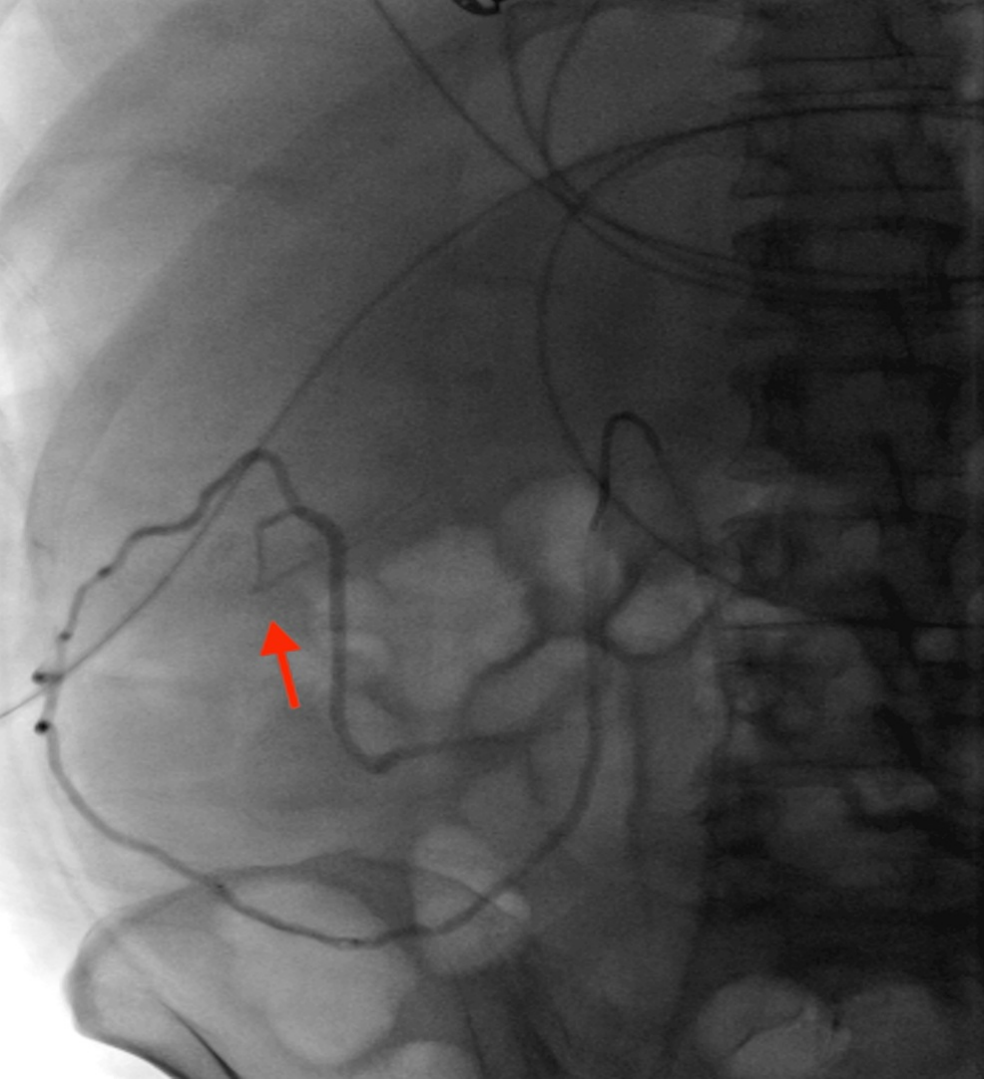
Colonic varix targeted for embolization.

**Figure 2:**
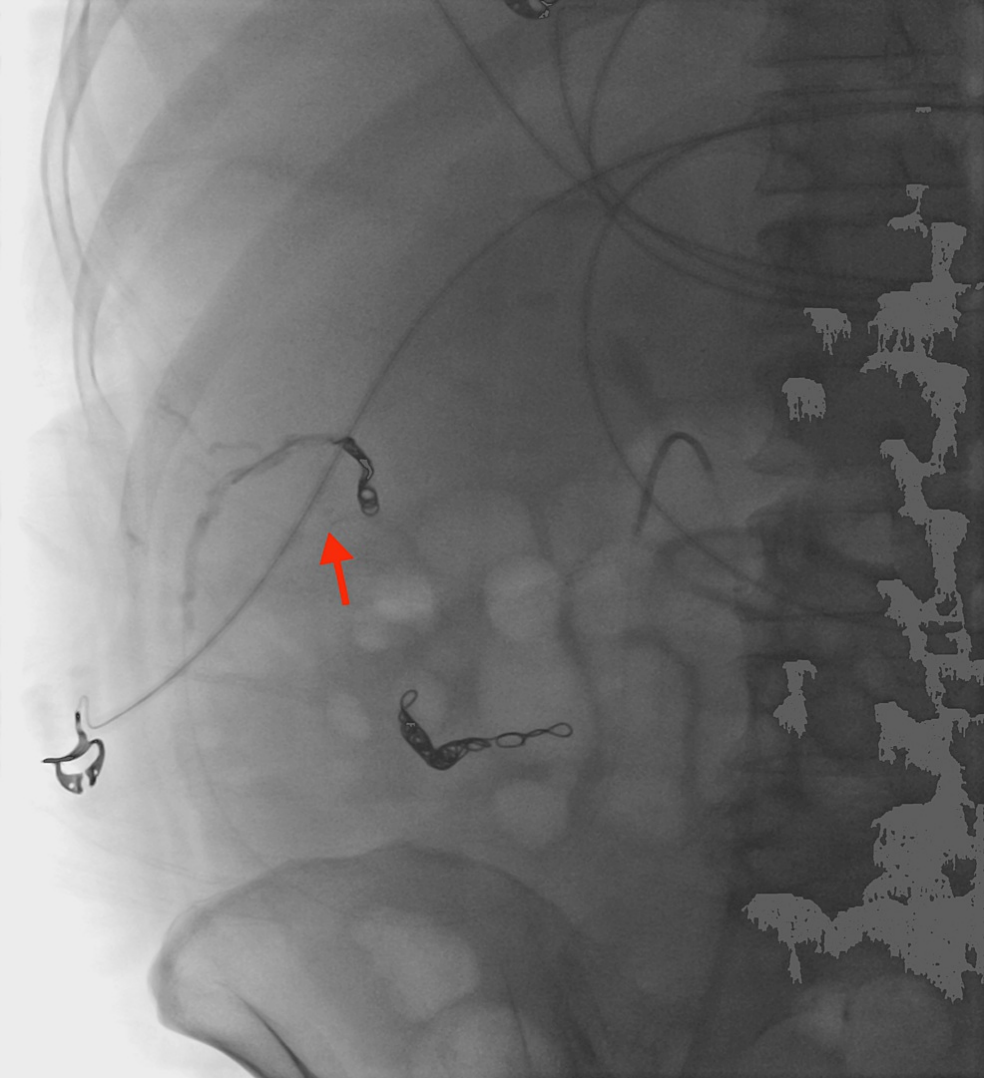
Embolization of colonic varix using coils and sotradecol foam.

**Figure 3:**
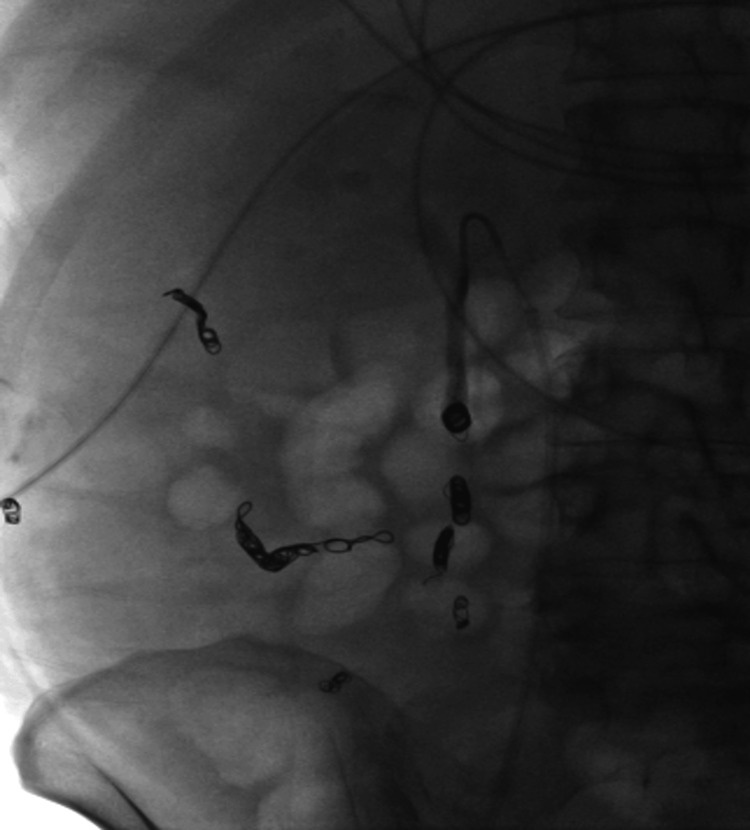
Post-procedure image showing no residual filling of colonic varices.

A 54-year-old female with alcohol-associated liver disease with cirrhosis and decompensation in the form of ascites, hepatic encephalopathy, and esophageal varices, with a Model for End-Stage Liver Disease (MELD) score of 23, Child-Pugh class C, was admitted with acute anemia after being found to have a hemoglobin of 6.5 g/dL (12.1 to 15.1 g/dL). Upon admission, she had an episode of melena followed by hematochezia and was started on ceftriaxone. Evaluation with esophagogastroduodenoscopy demonstrated non-bleeding grade 1 esophageal varices without stigmata of recent bleeding. Her melena resolved; however, she later developed hepatic encephalopathy and was started on lactulose and rifaximin. A few days later, she developed large-volume hematochezia with hypotension, requiring transfer to the intensive care unit. After resuscitation with 2 units of packed red blood cells, push enteroscopy and colonoscopy were performed and demonstrated fresh blood in the ascending colon and cecum with ascending colonic varices without evidence of active bleeding. There was bile seen throughout the extent of the insertion of the push enteroscope. The patient continued to have intermittent hematochezia; however, computed tomography angiography (CTA) of the abdomen and pelvis was not able to localize the exact source of bleeding. Transplant hepatology was consulted, and given the patient’s high MELD-Na score, acute hepatic encephalopathy, and lack of candidacy for liver transplantation due to continued alcohol use, transjugular intrahepatic portosystemic shunt (TIPS) placement was not recommended. After a multi-disciplinary discussion with interventional radiology (IR), transplant hepatology, and gastroenterology, the decision was made to proceed with empiric eradication of the colonic varices, which were felt to be the primary source of bleeding. IR performed venography and a coil-assisted retrograde transvenous obliteration (CARTO) of the ascending colonic varices by using a portosystemic shunt extending from the right gonadal vein, which connected to the varices. Figure 1 demonstrates one of the colonic varices that was targeted, and Figure 2 depicts the embolization of the varix. IR also performed proximal splenic artery embolization to decrease the overall portal pressure. No persistent colonic varices were noted post-intervention, as shown in Figure 3, and the patient had no further episodes of bleeding.

## Discussion

Ectopic varices are dilated portosystemic collateral veins located in unusual sites other than the gastroesophageal region and constitute 1% to 5% of all variceal bleeds in patients with portal hypertension [6]. Ectopic varices are clinically challenging due to difficulty in localization and a lack of standardized management, especially with bleeding. Colonic varices are a very rare cause of lower GI bleeding, with a reported incidence of 0.07%. These varices are typically associated with portal hypertension secondary to cirrhosis. While colonoscopy is the preferred method to detect colonic varices, the diagnostic yield is only about 68%, as the varices may be obscured by blood [7]. Notably, in our case, the colonic varices were especially difficult to appreciate during the colonoscopy, and this may have been due to concurrent octreotide infusion and hypotension during the procedure. Patients found to have colonic variceal bleeding may initially be managed medically with hemodynamic stabilization, transfusion, and antibiotics for the prevention of spontaneous bacterial peritonitis. No standardized management has yet been identified for colonic variceal bleeding, given the small cohort of patients with this complication. However, interventional procedures including colonic resection, TIPS, balloon-occluded retrograde transverse obliteration (BRTO), endoscopic variceal ligation, and coil embolization have been successfully performed [7-9]. Recently, modified versions of BRTO have been developed, such as coil-assisted retrograde transvenous obliteration (CARTO) and plug-assisted retrograde transvenous obliteration (PARTO). Upon review of the literature, there have been only five successful cases using BRTO or modified versions of the procedure in the treatment of colonic varices.

The first case, published in 2006 in Japan by Anan et al., described the first successful treatment of colonic varices by BRTO through the left renal vein in a patient with hepatic encephalopathy. This patient developed colonic varices on the splenic flexure of the descending colon [10]. In 2018, Matsumoto et al. performed BRTO through the right testicular vein using a microballoon catheter to prevent bleeding from ascending colonic varices. This was a preventative procedure performed on a patient with large nodular colonic varices that were not amenable to endoscopic intervention [11]. Liu et al. published the third successful case in 2020, in which BRTO via the right renal vein was performed to cease bleeding of ascending colonic varices located around the hepatic flexure [12]. In 2020, the first reported case of CARTO used for colonic varices was published by Maeda et al. The procedure was performed through the right renal vein and utilized both a microballoon catheter and two microcoils to obliterate the varices [13]. Most recently, in 2022, BRTO was used in the treatment of colonic variceal bleeding at a hospital in Qatar. In this case, a cirrhotic patient had lower gastrointestinal bleeding with no source identified on colonoscopy. Descending colonic varices were seen on the CT scan, and CARTO was performed using multiple embolization coils in conjunction with the balloon catheter [14]. 

## Conclusions

Very little data is available regarding the use of CARTO in treating colonic varices. However, our case report, in conjunction with the published data, serves as evidence that CARTO may be a useful alternative to TIPS or other invasive procedures in patients with colonic varices.
